# **The PrEP Laboratory Service Gap:** Applying Implementation Science Strategies to Bring PrEP Coverage to Scale in the United States

**DOI:** 10.1017/jme.2022.34

**Published:** 2022

**Authors:** Aaron Siegler, Patrick Sullivan

**Keywords:** Pre-exposure Prophylaxis (PrEP), Implementation Science, Insurance, Laboratory Testing

## Abstract

Using an implementation science framework, we detail how a national system for covering both standard and telemedicine laboratory testing would support a national PrEP program. Implementation strategies that will facilitate success include minimizing provider burden through uncomplicated billing systems and minimizing patient burden through centralized, online access systems. We anticipate that providing telemedicine and in-person laboratory testing options will optimize PrEP care by making it less burdensome, leading to cost-effective healthcare and improved population health.

## Introduction

HIV pre-exposure prophylaxis provides a new opportunity to address the HIV epidemic in the United States. There were over 227,000 persons using PrEP in 2019,^1^ yet there remains substantial opportunity for improvement with CDC estimating 1.1 million persons indicated for PrEP.^2^ Moreover, PrEP scale-up has not been in accordance with population need for HIV protection, with lower PrEP uptake among Black and Latino gay/bisexual men, women, and Black transgender women than their epidemic burden.^3^

Developing structural support for individuals to practice healthy behaviors is a foundation of public health practice. Clinically-based prevention interventions, such as preventive medications or disease screenings, are often underutilized. This is understandable, especially when people do not have symptoms, when the benefits of prevention behaviors are dependent on an unknown future probability of disease acquisition, and when there are substantial financial costs of clinical prevention services.

The need to optimize promotion of clinical prevention interventions drove the creation of the US Preventive Services Task Force in 1984, an independent panel that reviews and grades evidence for clinical prevention services.^4^ PrEP received the highest grade (A) from this panel, and the Affordable Care Act requires prevention services with this grade be covered with no cost-sharing (e.g., fees) by group health plans and health insurance issuers. This same guidance on coverage is also implemented for Medicaid expansion programs. Critically, guidance issued by the Departments of Health and Human Services (HHS) issued in 2021 clarified that coverage of PrEP services not only include the cost of medication, but also the cost of ongoing services requisite to care including quarterly clinician visits, required laboratory testing including for HIV and STD, and medication adherence counseling.^5^

Coverage of laboratory and clinician costs removes a central barrier to PrEP care, because laboratory costs of PrEP are high. Using the laboratory fee reimbursement schedule from CMS^6^ and CDC guidance^7^ regarding requisite labs for PrEP care among gay, bisexual, and other men who have sex with men, we found that a standard first-year set of PrEP laboratory tests was $1,013.10 (Appendix 1). This does not include the cost of four annual clinical visits, which adds substantial additional costs.

## PrEP Laboratory Services Coverage Gap

Despite the supportive USPSTF recommendation for PrEP, the no cost-sharing benefit does not apply to all persons in the United States. There remain substantial gaps that leave some of the persons hoping to use PrEP with substantial out-of-pocket costs, discouraging PrEP seeking and maintenance in care.

There are many ways to cover PrEP laboratory service gaps faced by many people in need of prevention services. We suggest developing a two-track system — in-person and telehealth care — that would allow the most users to initiate and remain on PrEP. Moreover, we do not anticipate that covering both options would increase the per user cost of the system; instead, telehealth services may potentially be cost-saving given that both lab and facility costs may be lower for telemedicine. Implementation science frameworks and change strategies should be incorporated into a national system to cover PrEP laboratory services.

For up to 48.9% of the US population, the ACA’s preventive services coverage and cost-sharing requirements are not federally required to be followed. Plans not federally required to follow CMS guidance include grandfathered healthcare plans (23.7 million persons),^8^ traditional, non-expansion Medicaid (59.8 million),^9^ Medicare Part D (48.0 million),^10^ and uninsured persons (31.2 million)^11^ (Appendix 2).

State Medicaid plans cover the cost of PrEP medication, but states may cover ancillary laboratory and clinician services at varying levels,^12^ resulting in a panoply of coverage rules. To our knowledge, there is no systematic source of data regarding which state Medicaid programs cover which services for PrEP care. Moreover, there is no systematic source of data regarding no-cost coverage for persons in grandfathered plans and for uninsured persons. In sum, no-cost sharing coverage is sufficiently fragmented and complex across this 48.9% of the US population that no systematic documentation of coverage has been made available to date.

An array of programs have been developed to fill gaps in PrEP ancillary services and drug coverage, including drug manufacturer assistance programs, state PrEP assistance programs, and usage of 340b drug program returns for service subsidy. There may be differential access to these services by place, program eligibility, and over time (e.g., as generics become more common, manufacturer programs may change). This changing landscape of reimbursement makes navigating these different coverage rules incredibly challenging. In fact, most large PrEP prescribers have dedicated “navigators” to help patients seek coverage for their PrEP care across the various sources of funding.

This patchwork system leads to considerable uncertainty for healthcare consumers and providers, likely deterring consumers from seeking PrEP and providers from prescribing it. In fact, PrEP usage is 99% greater in states that have expanded Medicaid and provide PrEP services coverage through state-level fee coverage programs that frequently feature PrEP navigation.^13^ Moreover, some of these sources of coverage might not be sustainable over time, potentially leading to future unexpected disruptions in PrEP care and lapses in protection from HIV infection.

The complexity of service coverage is even higher with the recent FDA approval of injectable cabotegravir.^14^ CDC’s updated clinician guidance on laboratory testing and eligibility assessment includes injectable cabotegravir,^15^ but how the medication and surrounding laboratory services will be covered by insurers is currently unclear. USPSTF has released a draft of systematic review questions that includes assessment of the benefits of injectable cabotegravir and other new PrEP regimens.

A national PrEP program, such as that proposed by Killelea and colleagues, could fill in PrEP laboratory coverage gaps, ensuring costs do not prevent those at risk from seeking PrEP care. Moreover, such a system could incorporate considerations of new PrEP regimens as they come to market. A recent cost-effectiveness analysis found that when PrEP drug costs are low, PrEP is a cost-saving intervention.^16^ This makes a national program a particularly attractive idea, because a likely outcome of such a program would be use of generic PrEP formulations that have low costs. Below, we detail a number of options for covering laboratory services nationally, using an implementation science framework to consider how the program might be developed and evaluated.

## Laboratory PrEP Program

A national PrEP services program, such as that proposed by Killelea and colleagues, will require laboratory support services that accommodate an array of patients and providers. We detail a patient-centered national service to cover PrEP laboratory services, proposing complementary models of in-person and telemedicine services to facilitate numerous PrEP access avenues. Using the Expert Recommendations for Implementing Change (ERIC) strategies,^17^ structures needed to support a national program are explored in Table 1.

**Table 1:** National PrEP Coverage Plan and ERIC Implementation Science Strategies to Facilitate Program Success

National Plan Component	ERIC Implementation Strategy	ERIC Examples
**Standard care laboratory and clinician PrEP service** *Summary*: National contract with major laboratory service providers to cover laboratory costs, and billing coverage of clinician service costs	Alter patients’ fees	HIV/STI/creatinine laboratory fees Clinician visit fees
	Make billing easier	Billing through existing Medicare/Medicaid channels, or other standard billing systems Clear guidance on billing procedures, with minimal paperwork
	Quality monitoring	Brief surveys of patients, providers, and administrators Ongoing stakeholder interviews
	Audit and feedback	Utilization metrics with performance targets, action plans if targets not met Equity assessment through population use measures such as PrEP-to-need ratio
	Educational outreach	Ensure patients are aware of no-cost PrEP options Outreach to clinicians and PrEP navigators regarding new coverage rules and implementation
**Telehealth laboratory and clinician PrEP services** *Summary*: National contracting for remote laboratory testing and telehealth visit provision	Change service sites	Home self-collection laboratory services Online clinician services
	Centralize technology assistance	Central website to facilitate access to telemedicine PrEP Portal for FAQ regarding telemedicine PrEP
	Alter patients’ fees	No-cost prepaid mailers for self-collection of specimens Specimens returned via prepaid mailer for standard laboratory testing at CLIA-certified laboratory
	Change records systems	Facilitate EHR transfer of results from selected laboratories to clinic records systems
	Educational outreach	Telemedicine services advertised online and at appropriate venues
	Quality monitoring	Brief surveys of patients, providers, and administrators Ongoing stakeholder interviews
	Make billing easier	Billing through national service contracts with select laboratories and provider networks
	Audit and feedback	Assessments of telehealth outcomes relative to standard care Equity assessment through population use measures such as PrEP-to-need ratio
	Facilitate data access	Standardized data collection for telemedicine PrEP to facilitate system-wide improvements, user tracking, and optimizing patient re-engagement in PrEP care after discontinuation

Standard care laboratory services could be covered with direct contracts with major laboratory services providers, or with a standard series of reimbursement payments for eligible laboratories. ERIC strategies to enhance provider prescription practices should include arrangements to ease billing by using existing channels and monitoring of service provision quality through ongoing surveys of patients and stakeholder interviews with providers to ensure services minimize provider burden. Audits should use PrEP utilization metrics and targets that feature not only assessments of overall use, but use according to equity. The PrEP-to-need ratio^18^ and other similar metrics allow for assessment PrEP equity relative to HIV epidemic burden. Such metrics have previously been used to demonstrate disparities in PrEP receipt among women,^19^ and geographic areas with lower income or higher concentrations of Black residents.^20^ Educational outreach to patients, providers, and PrEP navigators will facilitate increased use of the national program.

Telehealth laboratory services to promote PrEP are critical to provide primarily due to the high patient burden of four in-person clinic/lab visits per year, and to optimally serve those in need of PrEP who may be distant from PrEP providers such as those in rural areas.^21^ In ERIC terms, changing the default service site of PrEP care to one that is more convenient may improve both PrEP initiation and maintenance in care. CDC recommends for daily oral PrEP quarterly laboratory services to include HIV testing, 3-site gonorrhea/chlamydia testing (rectal, oral, urethral), and periodic creatinine monitoring.^22^ All are conducted with traditional laboratory testing, and are achievable with self-collection of specimens at home and mailing those specimens to laboratories for testing. These tests are currently being run by a lab under CLIA-based validation protocols,^23^ a procedure recommended by the CDC as a standard of care during the COVID-19 pandemic.^24^

In our studies of home care for PrEP, participants have successfully self-collected specimens for each of these tests, meeting CLIA validation performance for laboratory developed tests and with the procedure receiving high acceptability ratings from persons in PrEP care. We anticipate that remote testing would not add costs beyond standard laboratory testing; in our experience, the costs of remote laboratory testing for PrEP, including mailing and materials, are lower than standard laboratory fees.

ERIC strategies provide a number of insights into how to make a national telehealth laboratory program successful. To optimize telehealth benefits, online clinician services should be covered for persons otherwise facing a PrEP gap. A centralized program website could facilitate PrEP care initiation with referral to online providers that meet program and state medical licensing requirements. Systems to facilitate Electronic Health Records (EHR) transfer, from centralized mail-in laboratories to recipient clinics, would assist scale-up of telemedicine at traditional clinics that are interested in participating in the program.

Audits should ensure that telehealth services are being provided to the same standard as in-person PrEP services, and should incorporate equity metrics to ensure program scale-up to communities most in-need of PrEP care to optimize the societal prevention impact of PrEP provision.

## Discussion

There are many ways to cover PrEP laboratory service gaps faced by many people in need of prevention services. We suggest developing a two-track system — in-person and telehealth care — that would allow the most users to initiate and remain on PrEP. Moreover, we do not anticipate that covering both options would increase the per user cost of the system; instead, telehealth services may potentially be cost-saving given that both lab and facility costs may be lower for telemedicine. Implementation science frameworks and change strategies should be incorporated into a national system to cover PrEP laboratory services.

Future discussions and development of a national PrEP coverage system should consider these concepts and be informed by appropriate frameworks for implementation science to ensure a thorough exploration that seeks to minimize patient-level barriers to accessing PrEP.

## Notes

The research reported in this publication was supported by the National Institute of Mental Health R01MH114692 and by the Eunice Kennedy Shriver National Institute of Child Health and Human Development of the NIH (U19HD089881), under ATN protocol 159. The research was facilitated by the Emory Center for AIDS Research (P30AI050409). The content is solely the responsibility of the authors and does not necessarily represent the official views of the National Institutes of Health.

## Appendices

**Appendix 1:** Cost of First Year of Laboratory Testing Services for HIV Pre-exposure Prophylaxis

CPT Code	Test	Cost	Times per annum	Annual cost
87389	Hiv-1 ag w/hiv-1&2 ab ag ia	24.08	4	96.32
82565	Assay of creatinine	5.12	2	10.24
86803	Hepatitis c ab test	14.27	1	14.27
87340	Hepatitis b surface ag ia	10.33	1	10.33
86706	Hep b surface antibody	10.74	1	10.74
86704	Hep b core antibody total	12.05	1	12.05
86592	Syphilis test non-trep qual	4.27	4	17.08
87491	Chylmd trach dna amp probe (Rectal)	35.09	4	140.36
87591	N.gonorrhoeae dna amp prob (Rectal)	35.09	4	140.36
87491	Chylmd trach dna amp probe (Urethral)	35.09	4	140.36
87591	N.gonorrhoeae dna amp prob (Urethral)	35.09	4	140.36
87491	Chylmd trach dna amp probe (Pharyngeal)	35.09	4	140.36
87591	N.gonorrhoeae dna amp prob (Pharyngeal)	35.09	4	140.36
			**Total annual cost**	**1013.19**

Source: CMS 2021 Clinical Diagnostic Laboratory Fee Schedule.

**Appendix 2:** Persons Not Covered by USPSTF Rules against Cost Sharing

Plan type	Population in category (million)	Source of population estimate
Grandfathered^	23.7	"Final Rule on Grandfathered Health Plans Will Allow Higher Consumer Costs," Health Affairs Blog, December 14, 2020.
Traditional Medicaid, 2019 (Non-expanded Medicaid population)*	59.8	M. Guth, B. Corallo, R. Rudowitz, and R. Garfield, Medicaid Expansion Enrollment and Spending Leading up to the COVID-19 Pandemic, 2021.
Medicare Part D*	48.0	J. Cubanski and A. Damico, Key Facts about Medicare Part D Enrollment, Premiums, and Cost Sharing in 2021, 2021.
Uninsured	31.2	R.A. Cohen, E.P. Terlizzi EP, A.E. Cha, and M.E. Martinez, “Health Insurance Coverage: Early Release of Estimates from the National Health Interview Survey, 2020,” National Center for Health Statistics, August 2021.
Total	162.7	
Total US pop	332.5	United States Census Bureau Population Clock, 2022.
**Proportion of US population not covered by USPSTF rules against cost sharing**	**48.9%**	

Notes:

^ This population is not covered by USPSTF guidance: Kaiser Family Foundation

* These populations are not covered by USPSTF guidance: Health Affairs

## References

[1] P.S. Sullivan , C. Woodyatt , C. Koski , E. Pembleton , P. McGuinness , J. Taussig , A. Ricca , N. Luisi , E. Mokotoff , N. Benbow , A.D. Castel , A.N. Do , R.O. Valdiserri , H. Bradley , C. Jaggi , D. O’Farrell , R. Filipowicz , A.J. Siegler , J. Curran , and T.H. Sanchez , “A Data Visualization and Dissemination Resource to Support HIV Prevention and Care at the Local Level: Analysis and Uses of the AIDSVu Public Data Resource *,”* Journal of Medical Internet Research 22, no. 10 (2020): e23173. 33095177 10.2196/23173 PMC7654504

[2] D.K. Smith , M. Van Handel , and J. Grey , “Estimates of Adults with Indications for HIV Pre-exposure Prophylaxis by Jurisdiction, Transmission Risk Group, and Race/Ethnicity, United States, 2015,” Annals of Epidemiology 28, no. 12 (2018): 850–857. 29941379 10.1016/j.annepidem.2018.05.003

[3] A.J. Siegler , C.C. Mehta , F. Mouhanna , et al., “Policy- and County-Level Associations with HIV Pre-exposure Prophylaxis Use, the United States, 2018,” Annals of Eepidemiology 45 (2020): 24–31. 10.1016/j.annepidem.2020.03.013 PMC7246022 32336655

[4] H. Bauchner , P.B. Fontanarosa , and R.M. Golub , “JAMA Welcomes the US Preventive Services Task Force,” JAMA 315, no. 4 (2016): 351–2. 26813207 10.1001/jama.2015.18448

[5] Centers for Medicaid and Medicare Services (CMS), FAQs About Affordable Care Act Implementation Part 47: CCIIO OG MWRD 671. Accessed December 1, 2021, *available at* <https://www.cms.gov/CCIIO/Resources/Fact-Sheets-and-FAQs/Downloads/FAQs-Part-47.pdf> (last visited March 28, 2022).

[6] Centers for Medicaid and Medicare Services (CMS), “Clinical Laboratory Fee Schedule Files: CY 2021 Q4,” *available at* <https://www.cms.gov/medicaremedicare-fee-service-paymentclinicallabfeeschedclinical-laboratory-fee-schedule-files/21clabq4> (last visited March 28, 2022).

[7] Centers for Disease Control and Prevention *, US Public Health Service: Preexposure Prophylaxis for the Prevention of HIV Infection in the United States—2021 Update, A Clinical Practice Guideline*, *available at* <https://www.cdc.gov/hiv/pdf/risk/prep/cdc-hiv-prep-guidelines-2021.pdf> (last visited March 28, 2022).

[8] K. Keit , “Final Rule on Grandfathered Health Plans Will Allow Higher Consumer Costs,” *Health Affairs Blog*, December 14, 2020, *available at* <https://www.healthaffairs.org/do/10.1377/hblog20201214.170819/full/> (last visited March 28, 2022).

[9] Centers for Medicaid and Medicare Services (CMS), *Fact Sheet: Medicaid Facts and Figures*, *available at* <https://www.cms.gov/newsroom/fact-sheets/medicaid-facts-and-figures> (last visited March 28, 2022).

[10] J. Cubanski and A. Damico , “Key Facts about Medicare Part D Enrollment, Premiums, and Cost Sharing in 2021,” Kaiser Family Foundation, 2021, *available at* <https://www.kff.org/medicare/issue-brief/key-facts-about-medicare-part-d-enrollment-premiums-and-cost-sharing-in-2021/> (last visited March 28, 2022).

[11] R.A. Cohen , E.P. Terlizzi , A.E. Cha , and M.E. Martinez , “Health Insurance Coverage: Early Release of Estimates from the National Health Interview Survey, 2020,” *Health Insurance Coverage: Early Release of Estimates from the National Health Interview Survey, 2020*, August 2021, *available* at <https://stacks.cdc.gov/view/cdc/108816> (last visited March 28, 2022).

[12] Centers for Disease Control and Prevention (CDC), *Paying for PrEP*, *available at* <https://www.cdc.gov/hiv/basics/prep/paying-for-prep/index.html> (last visited March 28, 2022).

[13] See *supra* note 1.

[14] U.S. Food and Drug Administration (FDA), NDA 215499 Approval, *available at* <https://www.accessdata.fda.gov/drugsatfda_docs/appletter/2021/215499Orig1s000ltr.pdf> (last visited March 28, 2022).

[15] See CDC, *supra* note 7.

[16] J. McKenney , A. Chen , K.W. Hoover , et al., “Optimal Costs of HIV Pre-exposure Prophylaxis for Men Who Have Sex with Men,” PLoS One 12, no. 6 (2017): e0178170. 28570572 10.1371/journal.pone.0178170 PMC5453430

[17] B.J. Powell , T.J. Waltz , M.J. Chinman , et al., “A Refined Compilation of Implementation Strategies: Results from the Expert Recommendations for Implementing Change (ERIC) Project,” Implementation Science 10, no. 21 (2015). 10.1186/s13012-015-0209-1 PMC4328074 25889199

[18] A.J. Siegler , F. Mouhanna , R.M. Giler , et al., “The Prevalence of Pre-exposure Prophylaxis Use and the Pre-exposure Prophylaxis-to-Need Ratio in the Fourth Quarter of 2017, United States,” Annals of Epidemiology 28, no. 12 (2018): 841–849. 29983236 10.1016/j.annepidem.2018.06.005 PMC6286209

[19] A.J. Siegler , A. Bratcher , K.M. Weiss , F. Mouhanna , L. Ahlschlager , and P.S. Sullivan , “Location Location Location: An Exploration of Disparities in Access to Publicly Listed Pre-exposure Prophylaxis Clinics in the United States,” Annals of Epidemiology 28, no. 12 (2018): 858–864. 30406756 10.1016/j.annepidem.2018.05.006 PMC6261794

[20] A.J. Siegler , A. Bratcher , and K.M. Weiss , “Geographic Access to Preexposure Prophylaxis Clinics among Men Who Have Sex with Men in the United States,” American Journal of Public Health 109, no. 9 (2019): 1216–1223. 31318587 10.2105/AJPH.2019.305172 PMC6687234

[21] *Id.*

[22] *Supra* note 7.

[23] A.J. Siegler , K.H. Mayer , and A.Y. Liu , et al., “Developing and Assessing the Feasibility of a Home-Based PrEP Monitoring and Support Program,” Clinical Infectious Diseases: An Official Publication of the Infectious Diseases Society of America 68, no. 3 (2019): 501–504. 29982304 10.1093/cid/ciy529 PMC6336909

[24] Centers for Disease Control and Prevention (CDC), “PrEP During COVID-19,” available at <https://www.cdc.gov/nchhstp/dear_colleague/2020/dcl-051520-PrEP-during-COVID-19.html> (March 28, 2022).

